# THE MASON CARES STUDY: EFFECTS OF A STRESS MANAGEMENT INTERVENTION ON MALE FAMILY CAREGIVER BURDEN SCORES

**DOI:** 10.1093/geroni/igae098.0963

**Published:** 2024-12-31

**Authors:** Gilbert Gimm, Shannon Layman, Megumi Inoue, Emily Ihara, Catherine Tompkins

**Affiliations:** George Mason University, Fairfax, Virginia, United States; George Mason University, Fairfax, Virginia, United States; George Mason University, Fairfax, Virginia, United States; George Mason University, Fairfax, Virginia, United States; George Mason University, Fairfax, Virginia, United States

## Abstract

The Mason CARES study examined the association of a virtual stress management program on mean caregiver burden scores of 97 participants, including 16 male family caregivers of older adults with dementia. Using primary data collected from 97 participants, we analyzed family caregiver burden scores of male and female caregivers before and after implementation of a 9-week stress management program using Zoom sessions. Caregiver burden was measured using the Zarit Burden Interview (ZBI) score with a range from 0 to 48 (Bédard, 2001). Higher values (ZBI >=17.0) were associated with higher levels of burden. Among the 97 caregiver participants, 16% were men and 84% were women. Mean ZBI scores before and after the 9-week stress busting program intervention across the full sample decreased by 15.0% (from 24.1 to 20.5). However, the 9-week intervention was associated with a larger reduction in burden scores for male family caregivers than for female family caregivers. Specifically female family caregivers had a higher baseline ZBI score of 24.4 which decreased to 21.4 (p<.01). At the same time, male family caregivers had a lower baseline ZBI score of 22.5 which fell more sharply to 15.9 (p<.01). Findings show that male family caregivers of older adults living with dementia experienced a greater reduction in burden scores after the 9-week intervention compared to female family caregivers. These results suggest the need to continue recruiting men in stress management intervention studies to identify potential mechanisms that can help to explain gender differences associated with reducing family caregiver burden.

